# Latent progenitor cells as potential regulators for tympanic membrane regeneration

**DOI:** 10.1038/srep11542

**Published:** 2015-06-23

**Authors:** Seung Won Kim, Jangho Kim, Hoon Seonwoo, Kyung-Jin Jang, Yeon Ju Kim, Hye Jin Lim, Ki-Taek Lim, Chunjie Tian, Jong Hoon Chung, Yun-Hoon Choung

**Affiliations:** 1 Department of Burns and Plastic Surgery, Affiliated Hospital of Yanbian University, 1327 Juzi Street, Yanji, Jilin 133000, P.R. China; 2 Department of Rural and Biosystems Engineering, Chonnam National University, Gwangju 500-757, Republic of Korea; 3 Department of Biosystems & Biomaterials Science and Engineering, Seoul National University, Seoul 151-742, Republic of Korea; 4 Wyss Institute for Biologically Inspired Engineering at Harvard University, Boston, MA 02115, USA; 5 Department of Otolaryngology, Ajou University School of Medicine, San 5 Woncheon-dong, Yeongtong-gu, Suwon 443-721, Republic of Korea; 6 Department of Biosystems Engineering, Kangwon National University, Chuncheon 200-701, Republic of Korea

## Abstract

Tympanic membrane (TM) perforation, in particular chronic otitis media, is one of the most common clinical problems in the world and can present with sensorineural healing loss. Here, we explored an approach for TM regeneration where the latent progenitor or stem cells within TM epithelial layers may play an important regulatory role. We showed that potential TM stem cells present highly positive staining for epithelial stem cell markers in all areas of normal TM tissue. Additionally, they are present at high levels in perforated TMs, especially in proximity to the holes, regardless of acute or chronic status, suggesting that TM stem cells may be a potential factor for TM regeneration. Our study suggests that latent TM stem cells could be potential regulators of regeneration, which provides a new insight into this clinically important process and a potential target for new therapies for chronic otitis media and other eardrum injuries.

Tympanic membrane (TM) perforation is one of the most common clinical problems in the world. In particular, chronic otitis media resulting from TM perforations is a serious otologic disease that usually presents with conductive healing loss^1^. It has been widely accepted that most acute TM perforations spontaneously heal, as the TM has good regeneration capacity in the area around the annulus and malleus^1^,^2^. In contrast, chronic TM perforations usually do not heal without special surgical treatments such as an autologous graft from the muscle fascia or perichondrium^3^,^4^,^5^. Although these surgical treatments have shown a success rate of ~90% for healing chronic TM perforations in most studies, they also have several disadvantages, including 1) high cost of the operation; 2) defective donor sites; 3) the need for general or local anesthesia in some patients; 4) the need for complex microsurgical skills of the surgeons; and 5) aseptic procedures^3^,^4^,^5^.

Although the paper patch technique is still limited as the non-surgical alternative method to heal TM perforations, this method is frequently used in the clinics especially in the cases of acute TM perforations such as trauma. In this method, paper or biomaterial-based patches are used to guide TM cells to heal the lesions. Various biomaterial patches fabricated from collagen^6^, hyaluronic acid^7^, calcium alginate^8^, silk^4^,^9^, chitosan^4^,^5^,^10^, AlloDerm^2^, or urinary bladder matrix^11^, have been studied as alternatives to surgical repair for acute or chronic TM regeneration. In addition, some drugs (e.g., basic fibroblast growth factor and pentoxifylline) and tissue engineering techniques have also been studied^8^,^9^,^12^,^13^,^14^. Although these studies showed some potentials for acute TM regeneration, it should be noted that no definite treatment alternatives to surgery for chronic TM perforations have been reported until now.

To address this challenge, here, we propose a novel approach for regeneration of the TM in both acute and chronic TM perforations without surgical treatments. We focused on the role of latent stem cells during regeneration of TM; we speculated that TM regeneration began near the peripheral annulus and the malleus in TM perforations^1^,^2^,^3^,^4^,^5^,^10^, possibly because of the presence of latent progenitor or stem cells within the epithelial layers of the TM. This study was composed of two parts: 1) identification of TM stem cells using epithelial stem cell markers; 2) investigation of the distribution of TM stem cells in the normal TM and in the TM during regeneration in both acute and chronic perforations. Strikingly, we found that TM stem cells regulated TM regeneration in both acute and chronic perforations, demonstrating a potential therapy for TM rupture and full hearing restoration.

## Results

### Isolation of TM stem cells

**Figure 1:**
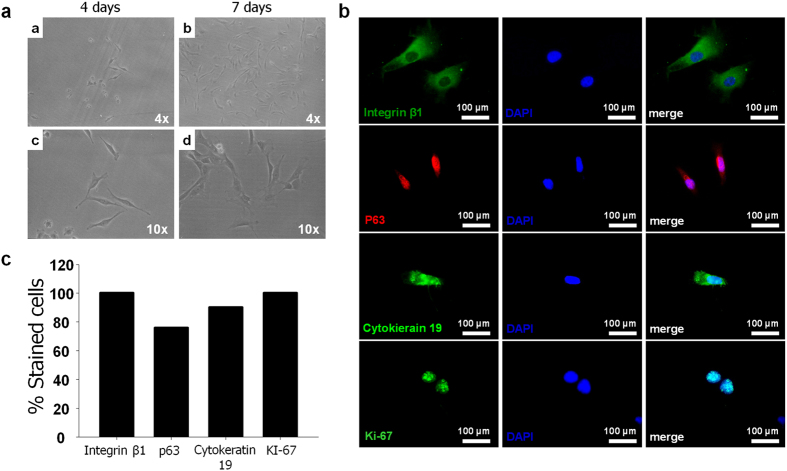
Isolation of stem cells from TMs. **a**) Optical microscopy (Magnification x4, x10). (**b**) Immunocytochemistry of epithelial stem cell markers, INGβ1, CK19, p63, and proliferation marker, Ki67. (**c**) Proportion of TM stem cells expressing INGβ1, CK19, p63 and Ki67.

To explore the role of TM stem cells as a regulator in TM regeneration, potential stem cells were collected from TMs of Sprague-Dawley (SD) rats. In primary cultures, adherent isolated TM stem cells were observed after 4 and 7 days (Fig. 1). Compared with the 4-day culture, the spindle-shaped cells in the 7-day culture showed an increased cell number as a result of increased proliferation, and the observed growth was relatively rapid.

Although no optimal marker for TM stem cells is currently available, it has been accepted that the keratinized epithelium of the TM is similar to the epidermis of regular skin^15^,^16^. We thus hypothesized that markers of epidermal stem cells could be used to identify potential TM stem cells in this study. The immunocytochemical results for the isolated cells are shown in Fig. 1b. Epithelial stem cell markers (Cytokeratin 19 (CK19), Integrin β1 (INGβ1), and p63) and the proliferation marker Ki-67 were used to identify stem cells in the TM. We determined the proportion of cells expressing INGβ1, p63, CK19, and Ki67 as 100%, 76%, 90%, and 100%, respectively (Fig. 1c). These positively stained cells were thus considered to be potential stem cells in the TMs and expected to be potential regulators for TM regeneration.

### Distribution of potential stem cells in normal rat TM

In previous studies, our group developed animal models of acute and chronic TM perforation^4^,^5^,^10^,^17^. In the previous studies, we observed that some TMs in animals with acute or chronic TM perforations spontaneously regenerated within a few weeks (after preparation of the models) and most of the TM regeneration began from the region around the annulus and malleus. We thus suspected that a potential unknown regulator near the annulus and malleus influences regeneration. As a potential regulatory candidate, we speculated that latent stem cells are present in these generative loci.

**Figure 2:**
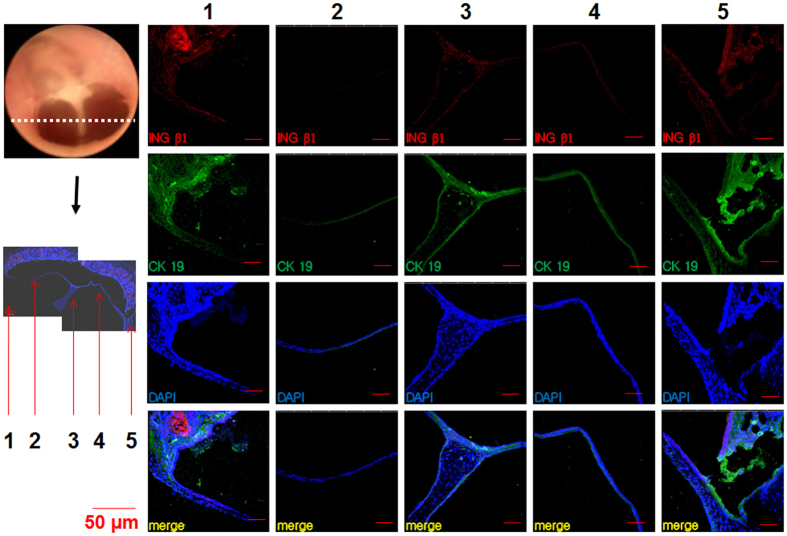
Immunostaining of normal rat TM sections with INGβ1 and CK19. Malleus handle (3), annulus (1, 5), pars tensa (2, 4).

We first investigated the existence and distribution of TM stem cells, as indicated by epithelial stem cell markers, in normal control TMs. We prepared cryo-sections of normal rat TMs and immunostained them to evaluate the expression of INGβ1 and CK19 (Fig. 2), which showed higher expression than P63 (Fig. 1c). The normal TMs presented positive staining for the stem cell markers in all areas of the membrane; however, staining was much more prominent around the malleus handle and annulus than in the pars tensa (Fig. 2). In addition, we confirmed that the staining intensity of CK19 was stronger than that of INGβ1 in normal TMs (Fig. 2). Our findings suggest that stem cells exist in the normal TM, predominantly in previously identified generative regions.

### Distribution and spontaneous regeneration potential of stem cells in acute TM perforations

**Figure 3:**
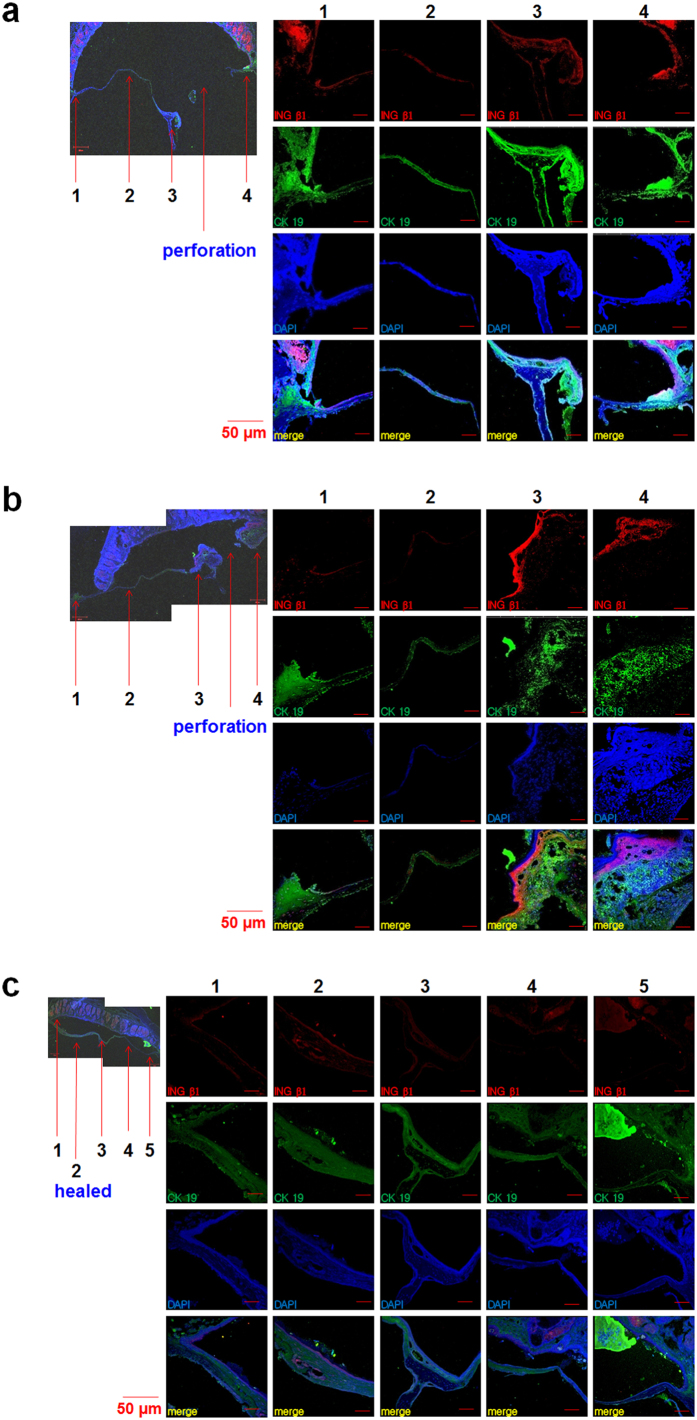
Expression of INGβ1 and CK19 in acute TM perforations. (**a**) TM at the first day after perforation, (**b**) TM at the fifth day after perforation. (**c**) TM at the tenth day after perforation. 1,5; annulus, 3; malleus handle, 2; pars tensa, 4; perforation site.

We analyzed the distribution of stem cells during regeneration of TMs after acute perforation using immunohistochemical staining for INGβ1 and CK19. After the TMs were perforated, we observed significantly higher expression of the epithelial stem cell markers INGβ1 and CK19 in all areas (Fig. 3a) compared to normal TMs (Fig. 2). The expression of INGβ1 and CK19 was more intense around the malleus handle and the annulus areas near the perforation holes (perforation sided annulus) than in the other areas (Fig. 3a). At 5 days after acute perforation, we observed that the lesion size was decreasing and the epithelial thickness was larger than that observed initially at 1 day, indicating that TM regeneration began spontaneously and immediately in acute perforations (Fig. 3b). Compared with the day 1, we observed that expression of INGβ1 and CK19 was significantly decreased in the non-perforated regions including the pars tensa and the annulus, whereas expression was still high in the perforated regions (Fig. 3b). This finding indicates that the regeneration potential was higher around perforation sites including the malleus handle and annulus areas. At 10 days, the TMs were completely healed and the expression of INGβ1 and CK19 was significantly reduced to a similar level to that in normal TMs (Fig. 3c).

### Distribution and spontaneous regeneration potential of stem cells in chronic TM perforation

**Figure 4:**
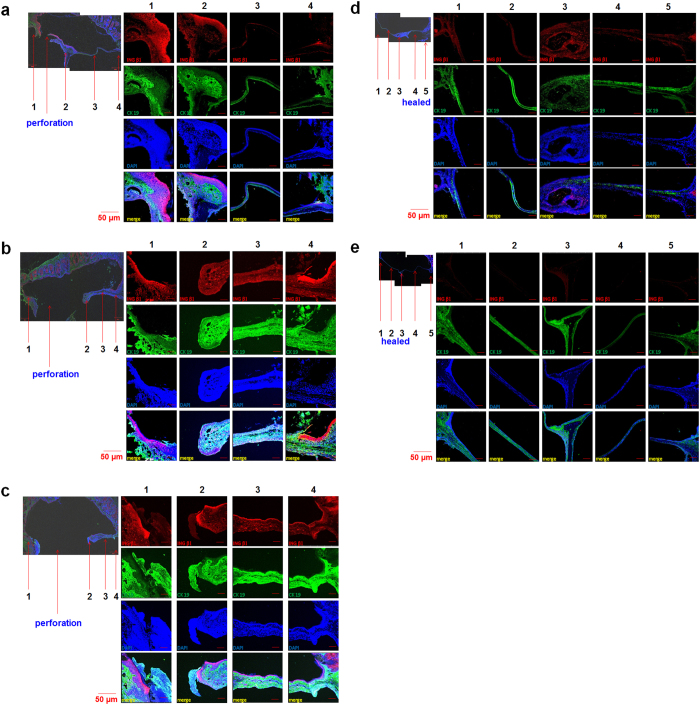
Expression of INGβ1 and CK 19 in chronic TM perforations. **a**) TMs with perforations showed high expression of INGβ1 and CK 19 on the first day, (**b**) 1 week, (**c**) 3 weeks, (**d**) 6 weeks, (**e**) 9 weeks after completing the whole 7-day procedure for chronic perforations. The completely healed TMs showed low expression of INGβ1 and CK 19 at 6 weeks and 9 weeks after the chronic perforation procedure. 1, 5; annulus, 3; malleus handle, 4; pars tensa, 2; perforation site.

As shown in Fig. 4, on the first day after the 7-day procedure for chronic perforation was complete, INGβ1 and CK19 were strongly expressed in the remnant TM around the malleus handle and the perforation side of the annulus compared to normal TMs. Additionally, these markers were relatively weakly expressed in the pars tensa and non-perforation side of the annulus (Fig. 4a). However, the staining intensities of INGβ1 and CK19 were much higher in all areas including the malleus handle and even the pars tensa and non-perforation side of the annulus on the first, third, sixth, and ninth week compared to normal TMs and TMs on the first day after the chronic perforation procedure. Before these experiments, we expected that the chronically perforated TMs would show minimal regeneration with faint expression of epithelial stem cell markers. However, the expression of INGβ1 and CK19 was high even at 9 weeks after the chronic perforation procedure. Only the expression in the pars tensa and non-perforation side of the annulus appeared to be lower than that in samples from other days. This difference appeared to reflect the healing state of the TM. The TM on the third week (Fig. 4f) showed almost complete healing, in contrast to the other TMs (Fig. 4b,d,e). Most TMs with large perforations showed high regeneration potential even 9 weeks after the chronic perforation procedure.

The spontaneously regenerated TMs in animals with chronic TM perforation showed significantly decreased expression of stem cell markers (Fig. 4f,g) to levels similar to those in the normal TM or regenerated TM in the acute TM perforation model (Figs. 3 and 4). We therefore speculated that latent stem cells regulate TM regeneration in chronic perforation, as observed in acute perforation.

### Stem cell marker expression in the TM

**Figure 5:**
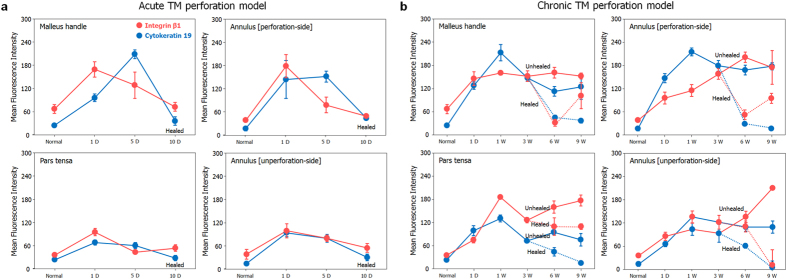
The intensity of expression on potential stem cell makers (Integrin β1 (red color) and Cytokeratin 19 (blue color) according to the anatomic areas (malleus handle; annulus (perforation site); pars tensa; annulus (unperforation site) in the TMs with acute or chronic perforations. **a**) Acute TM perforation model. (**b**) Chronic TM perforation model.

We quantitatively analyzed and compared the expression intensity of INGβ1 and CK19 in landmarked locations in the TM at different time points (Fig. 5). Normal and healed TM, regardless of acute or chronic perforation, showed low expression of the epithelial stem cell markers INGβ1 and CK19 in all areas of the TM. However, during the acute or chronic perforation state, INGβ1 and CK19 were up-regulated, especially in the areas around the malleus handle and the perforation side of the annulus. We also observed that expression of CK19 was much stronger and more sensitive than that of INGβ1, depending upon the TM state.

## Discussion

Our results support the notion that the latent stem cells are key regulators of TM regeneration in acute and chronic perforations. The epithelial layer of the TM is similar to the epidermis of the skin^16^. Although further studies of TM stem cell markers are required to identify the exact stem cells in the TM, we demonstrated that potential stem cells in the TM present high expression of epithelial stem cell markers and proliferation markers. Stem cells are responsible for tissue repair and the high proliferation of epithelial tissues^18^. A broad distribution of potential stem cells was observed in both normal TMs and perforated TMs. After acute TM perforation, epidermal stem cell markers were significantly upregulated. The epidermal stem cell markers INGβ1 and CK19 were highly expressed during the period of perforation; however, their expression decreased to normal levels after the perforations healed. In the unhealed perforations in animals with chronic perforations, the increased expression of INGβ1 and CK19 remained, even at 9 weeks. A similar trend for the expression of TM stem cells in the acute and chronic perforations was observed; strong expression of stem cell markers was observed in the perforated regions, whereas the expression was significantly decreased when the TMs were regenerated. In the normal human TM, possible progenitor cells have been found in the malleus and annulus region^19^; this observation is similar to our findings in rats, where marked upregulation of INGβ1 and CK19 was observed around the malleus handle and annulus after TM perforation. These observations provide the important insight that TM stem cells continuously act on the TM surface, especially during both acute and chronic TM regeneration, as a key regulator (see Fig. 5 for a summary), and thus provide a novel target for regeneration of the TM without surgical intervention. We therefore considered that these latent stem cells could play a critical role in the acute TM regeneration mediated by chitosan patches that we reported previous^4^,^5^ and also in a rat chronic TM perforation model of otitis media. Together, these observations provide the important insight that TM stem cells continuously act on the TM surface, especially during both acute and chronic TM regeneration, as a key regulator (see Fig. 5 for a summary), and thus provide a novel target for regeneration of the TM without surgical intervention.

Our results suggest that the latent progenitor or stem cells within epithelial layers in TMs may play an important role as a regulator of regeneration in both acute and chronic perforations, and that controlling stem cells in the perforated TMs may be a potential clinical therapy for TM regeneration without surgical treatments. The potential stem cells in TMs presented highly positive staining for epithelial stem cell markers. The distribution of epithelial stem cells was consistent across normal TMs, perforated TMs, and regenerated TMs, providing strong evidence that these stem cells may regulate TM regeneration. In the normal TM, the epithelial stem cells were present at low levels in all areas. In contrast, TM potential stem cells were present at high levels in the perforated TM, especially near the lesions, regardless of whether they were acute or chronic. The high sensitivity of the TM cells to stem cell growth factors could possibly be leveraged to generate new therapeutic modalities for TM regeneration. Epithelial stem cells as a regulator of TM regeneration may provide new insight for understanding regeneration mechanisms and developing potential therapies. In summary, we report that TMs contain progenitor or epidermal stem cells in areas close to the malleus handle and annulus, and these cells may be key regulators of TM regeneration. This innate stem cell-based TM regenerative capacity may be harnessed and augmented by our proposed therapeutic approach and thus reduce the need for invasive, less effective and potentially dangerous surgical interventions.

## Methods

### Materials

Thirty five SD female rats (Orient Bio Inc., Gyeonggi-Do, Korea) were used in this study. The Ajou University School of Medicine - Institutional Animal Care and Use Committee (AUSM-IACUC) approved the surgical procedures in accordance with the guidelines regarding the care and use of animals for experimental procedures. All efforts were made to minimize the number of animals used and their suffering.

### Primary TM cell culture

Five SD rats (3 weeks) were decapitated under deep anesthesia of Zoletil 50 (Virbac Laboratories, Carros, France) and 2% Rompun (Bayer Korea, Ansan, Korea), and the TMs were isolated. To obtain TM-derived cells, TMs were washed 2 times with phosphate-buffered saline (PBS) containing 100 U/ml penicillin and 100 μg/ml streptomycin, and cells were isolated by 0.05% trypsin-EDTA (Gen DEPOT) digestion for 10 min at 37 °C or enzymatic digestion using 0.66% collagenase type II (GIBCO BRL, Burlington, Ontario, Canada) for 4 h at 37 °C. After mechanical agitation, cells were passed through 100-μm cell strainer (BD Falcon, Erembodegem, Belgium) and cultured in α-minimum essential medium (α-MEM; Gibco BRL, Gaithersburg, MD, USA) supplemented with 10% fetal bovine serum (FBS; Gibco, Milan, Italy), 100 U/ml penicillin, and 100 μg/ml streptomycin at 37 °C in 5% CO_2_.

### Immunocytochemistry

Cells in the fourth subculture were washed with PBS 2 times and fixed in 4% paraformaldehyde for 10 min at room temperature. Permeabilization was accomplished using 0.2% Triton X-100 for 5 minutes, followed by blocking in 15% FBS for 1 h. Cells were incubated with primary antibodies against cytokeratin 19 (CK19) (Millipore, MA, USA), integrin β1 (INGβ1), p63 and Ki-67 (Abcam, Cambridge, MA, USA) and then the antibodies were detected by FITC- or Cy3-conjugated secondary antibodies (Jackson, ImmunoResearch Laboratories). The nuclei were stained with 4′,6-diamidino-2-phenylindole (DAPI, 10 mg/ml; Sigma Aldrich). Immunofluorescence was imaged by confocal microscopy (Carl Zeiss, Thornwood, NY, USA). The percentage of positively stained cells was calculated as: the number of FITC or Cy3/DAPI colabeled cells × 100/total number of DAPI-stained cells.

### Classification of TM perforations in rats

To determine the stem cell distribution in TMs, 30 SD rats (8 weeks, 180–230 g) were assigned to an acute TM perforation group (ATMP, n = 9), a chronic TM perforation group CTMP, n = 18), and a control group (n = 3).

Acute TM perforations (ATMP) were prepared by mechanical injury using a micropick under a surgical microscope (4, 5, 10). The perforation size was approximately 50% of the TM size. It is noted that we used bilateral TMs in rats for this work. The TMs in the ATMP group were harvested on post day 1 (n = 6), day 5 (n = 6) and day 10 (n = 6), respectively. Chronic TM perforations were prepared by Choung’s COM model I (17). In the CTMP group, the TMs were harvested on post day 1 (n = 6), day 7 (n = 6), day 21 (n = 6), day 42 (n = 6), day 63 (n = 6) and day 126 (n = 6), respectively. Among the prepared chronic TM perforation animals, 5 chronic TM animals (20.8%) were spontaneously healed within 3 weeks or 6 weeks. These closed TMs were harvested and stained at 6 weeks and 9 weeks, respectively. TMs in the control group were also harvested, and immunofluorescence staining was performed on all TM groups to determine the stem cell distribution.

### Immunohistochemistry

Immunohistochemistry was performed to determine the potential stem cell distribution. Following tissue processing and OCT compound (Sakura Finetek Inc.) embedding, sections were cut at 10 μm and mounted onto Probeon Plus microscope slides (Fisher Scientific, Pittsburgh, PA, USA). Slides were incubated at 37 °C for 1 hour. The staining procedure was performed at room temperature in a light-tight humidified staining box under low-light conditions. Briefly, 10-μm sections were rinsed in 1× PBS and then blocked with 1% bovine serum albumin (BSA)/0.2% Triton X-100 for 30 min. The slides were then incubated with mouse anti-rat CK19 monoclonal antibody (Chemicon International, CA, USA) and rabbit monoclonal INGβ1 antibody (Abcam, USA) overnight at 4 °C, then washed with of 0.5% BSA in PBS two times. Subsequent incubation with the corresponding secondary antibody (1:200) was performed for 60 min followed by two washes in 0.5% BSA in PBS. Finally, cell nuclei were counterstained with DAPI for 5 min, washed twice with 0.5% BSA in PBS, cover slipped in an anti-fade mounting medium, and sealed with nail varnish. Under confocal laser scanning microscopy (LSM710, Carl Zeiss, Jena, Germany), images of various parts of the TM were acquired. The fluorescence intensities of five adjacent points were calculated on each site of the samples with Photoshop program. The fixed size pixels placed randomly at 10 or more different regions within the landmarked location in the TM, and each of the fluorescence intensities was averaged.

## Additional Information

**How to cite this article**: Kim, S. W. *et al.* Latent progenitor cells as potential regulators for tympanic membrane regeneration. *Sci. Rep.* **5**, 11542; doi: 10.1038/srep11542 (2015).
